# Mr. Magin's Case of Cutaneous Affection

**Published:** 1802-12-01

**Authors:** T. Girdlestone

**Affiliations:** Yarmouth


### *To the Editors of the Medical and Phyſical Journal.*

GENTLEMEN,

ABOUT two years ago, two brothers of the name of Lambert, were ſhown to me, who came from a diſtant part of Suffolk with a very extraordinary appearance, which came on in a few weeks after they were born, and had ever ſince remained extended over the ſkin, except on the face, palms of the hands, and ſoles of the feet, which were almoſt the only parts that were ſmooth and natural.

This deformity was a horny ſort of covering, which ſeemed moſt nearly to reſemble an innumerable body of warts of a brown colour and cylindrical figure, riſing to a uniform height, of about half an inch, and growing as cloſe as poſſible to each other, but ſo ſtiff and elaſtic, that when the hand was drawn over them they made a ruſtling noiſe. Theſe young men ſaid, that they had a brother in the ſame manner, and their father and grandfather had the ſame ſort of ſkin; but, if I recollect rightly, that their ſiſter or ſiſters were free from this deformity. Theſe men were perfectly healthy and good looking; one was under twenty, and the other not much older; but their complexions were unuſually florid. Theſe were all the particulars that a few minutes converſation with them enabled me to notice; and I did not know till I afterwards read the ſecond part of Dr. Willan's valuable work on the Diſeaſes of the Skin, that the caſe of Lambert the grandfather, and Lambert the father of theſe young men, had been ſo accurately detailed by Mr. Machin in 1731, and by Henry Baker, Eſq. in 1755, in the Philoſophical Tranſactions, as quoted by Dr. Willan, Order II. iv. Icthyoſis; or I ſhould have enquired more minutely how far all the circumſtances which are related by theſe gentlemen correſponded with the deformity of this third generation of Lambert's. But perhaps ſome of your readers, who reſide in the neighbourhood of theſe men, may be able to ſupply my defects on this curious race.

The following deſcription of a deformity of the ſkin, with the encloſed drawing, was lately ſent me by Mr. Magin, Surgeon of the Plymouth Diviſion of Marines, with permiſſion to have it publiſhed, ſhould you think it worthy of a place in your very uſeful Journal. I am, &c.

*Yarmouth, Oct.* 25, 1802. T. GIRDLESTONE, M. D.

---

WILLIAM BUDD, aged twenty-four, lately diſcharged from the Plymouth diviſion of marines, of a pretty ſtrong make and healthy conſtitution, has on his back ſeveral large pendulous tumours tumours, the chief of which are ſketched in the annexed plate. The firſt tumour on the nape of the neck is larger than an ordinary man's fiſt, thoſe underneath are ſtill larger. They are flaccid, pendulous, and containing (if we may judge by the feel) a fatty ſubſtance; and from the ſenſibility of their ſkin and their vaſcular appearance, they are well ſupplied with nerves and blood veſſels, excepting at particular points, where white ſpots, and two or three knobs are obſervable. Theſe ſpots, by his own account, have been the ſeat of ſmall ulcers, from bruiſes, friction, or ſome kind of external injury. The tumours are of a chocolate colour and without hair. But the upper part of the nape of the neck, the back, the left buttock and loins, and the whole of the left arm to the elbow, together with the left ſide, are of the ſame colour, and covered over with a thick coat of ſtrong black hair.

The top of the right ſhoulder, ſome way down the humerus, is diſcoloured, and covered with hair in the ſame manner; but the remaining part of the right ſide is of a natural colour, and the ſkin is fair and delicate. The lower extremities are alſo diſcoloured in a variety of points, each in extent of ſurface from the breadth of a ſhilling to a crown-piece, and thickly tufted with hair.

His parents are healthy, without any corporeal blemiſh, and his mother, he ſays, uſed to account for theſe appearances on his back, &c. by ſaying, that ſhe longed for two hares while ſhe was pregnant with him. The tumours do not appear to have injured his general health, and they have gradually increaſed with the body in ſize. The only inconveniency he ſuffers from them is in warm weather, when the perſpiration is apt to fret the parts, and produce excoriations or ſmall ulcers, which ſoon heal up. In the courſe of ſurveys of men here, in conſequence of the reduction of troops, we have met with many caſes of luſus naturæ, but none ſo ſtriking as the above caſe. We have alſo met with ſeveral ſubjects in whom part of the hair of the head, eye-brows, &c. has been changed from the natural colour to a perfect white, producing a pyebald appearance, and generally in men who have ſerved in the Weſt Indies during the greater part of the late war, who attribute theſe changes to the yellow fever, under which moſt of them have laboured two or three times.

